# Molecular epidemiology and pathogenic potential of *mcr-1*-positive *Escherichia coli* isolated from healthy and diseased poultry in Jiangxi, China

**DOI:** 10.3389/fmicb.2026.1855169

**Published:** 2026-07-07

**Authors:** Wan-Quan Liu, Jia Tan, Jiang-Nan Huang, Hua-Yuan Ji, Hai-Qin Li, Quan-Yong Zhou, Chen-Long Liu, Qing-Bu Mei, Mei-Fang Tan

**Affiliations:** 1 School of Basic Medicine, Qiqihar Medical University, Qiqihar, China; 2 Key Laboratory of Drug Food Homologous Resources and Metabolic Disease Prevention and Treatment in Heilongjiang Province, Qiqihar, China; 3 Institute of Animal Husbandry and Veterinary Science, Jiangxi Academy of Agricultural Sciences, Nanchang, China; 4 Jiangxi Provincial Key Laboratory of Green and Healthy Breeding of Livestock and Poultry, Nanchang, China

**Keywords:** APEC, colistin resistance, *Escherichia coli*, Jiangxi Province, *mcr-1*, multidrug resistance, poultry

## Abstract

**Introduction:**

The emergence and dissemination of *mcr-1* has raised global public health concerns, and poultry is a major reservoir. However, systematic investigations of *mcr-1*-positive *Escherichia coli* in Jiangxi Province, an important poultry production base in southern China, remain limited.

**Methods:**

From 2020 to 2024, 591 *E. coli* strains were collected from diseased (*n* = 291) and healthy (*n* = 300) poultry across 11 cities in Jiangxi Province. *mcr-1*-positive strains were identified by colony PCR. The minimum inhibitory concentrations were determined for 16 antimicrobial agents. Whole-genome sequencing and bioinformatics analysis were conducted. Pathogenicity was assessed using the *Galleria mellonella* infection model.

**Results:**

Among the 591 strains, 24 were *mcr-1*-positive, including 15 avian pathogenic *E. coli* (APEC) strains from diseased poultry and 9 intestinal *E. coli* strains from healthy poultry. All 24 isolates exhibited colistin resistance and 100% multidrug resistance. High resistance rates were observed for apramycin (100%), sulfisoxazole (100%), ampicillin (95.83%), ceftiofur (95.83%), tetracycline (95.83%), and sulfamethoxazole-trimethoprim (95.83%), whereas all isolates remained susceptible to meropenem. Genomic analysis identified a diverse array of acquired resistance genes, including *tet(A)*, *bla*_CTX-M_, *sul2*, and *floR*, consistent with the phenotypic resistance profile. *mcr-1* was predominantly located on IncI2 plasmids, with chromosomal integration detected in eight APEC isolates. Virulence genotypic analysis revealed high prevalence of key APEC-associated genes (*iss*, *ompT*, *hlyF, iroN*, *iutA*), whereas *iucD* and *papG* were absent in all isolates. The infection assay demonstrated strong lethality in most isolates, with some intestinal *E. coli* from healthy poultry also exhibiting high mortality rates.

**Discussion:**

The coexistence of plasmid-borne and chromosome-integrated *mcr-1* genomically suggests dual maintenance mechanisms with potential transmissibility. Intestinal *E. coli* from healthy poultry carried higher resistance burden and virulence potential, revealing a hidden reservoir. These findings suggest the need for expanded surveillance, prudent antimicrobial use, and functional validation to further assess the potential dual threat to animal and public health.

## Introduction

The emergence and global dissemination of mobile colistin resistance (*mcr*) genes have raised significant public health concerns, as colistin serves as a last-resort antibiotic for treating multidrug-resistant Gram-negative bacterial infections (Liu et al., 2016; Li et al., 2024). Among the ten identified *mcr* variants, *mcr-1* is the most prevalent and has been detected worldwide in *Escherichia coli* isolates from humans, animals, and the environment (Xiaomin et al., 2020). The plasmid-borne nature of *mcr-1* facilitates its horizontal transfer across different bacterial species and genetic backgrounds, accelerating the spread of colistin resistance (Hu et al., 2023). In China, the use of colistin as a growth promoter in livestock was banned in 2017; however, *mcr-1*-positive *E. coli* strains continue to be detected in humans, food-producing animals, food products, and the environment, suggesting the persistence of this resistance determinant even in the absence of selective pressure (Hu et al., 2023; Lu et al., 2023).

Poultry is considered a major reservoir of *mcr-1*-positive *E. coli*, and transmission from poultry to humans through the food chain has been well documented (Liu et al., 2016; Trung et al., 2017; Wang et al., 2025). Avian pathogenic *E. coli* (APEC), a leading pathogen causing colibacillosis in poultry, causes systemic infections including airsacculitis, pericarditis, and perihepatitis, resulting in substantial economic losses worldwide (Nawaz et al., 2024). Moreover, APEC strains frequently exhibit multidrug resistance (MDR), which severely complicates clinical treatment and disease control strategies (Jamali et al., 2024). In contrast, intestinal *E. coli* colonize the gastrointestinal tract of healthy poultry without causing clinical disease, yet they act as important reservoirs of antimicrobial resistance (AMR) genes that may be transferred to pathogenic strains or directly to humans (Massella et al., 2021). Intestinal *E. coli* from healthy poultry constitute a hidden reservoir of AMR and resistance genes, posing a concealed threat of transmission through the food chain, direct contact, and environmental contamination (Li et al., 2025). Therefore, clarifying the genomic and phenotypic characteristics of *mcr-1*-positive *E. coli* from poultry is critical for evaluating the public health risks associated with commercial poultry production.

Jiangxi Province is an important poultry production base in southern China, characterized by a large breeding scale and high stocking density. However, the prevalence and molecular characteristics of *mcr-1*-positive *E. coli* in this region have not been fully elucidated. Our previous epidemiological studies have demonstrated that bacterial pathogens are widely distributed in poultry from Jiangxi, and the AMR of these strains has become increasingly severe (Tan et al., 2023; Tan J. et al., 2025; Tan M. F. et al., 2025). Nevertheless, systematic investigations of *mcr-1*-positive *E. coli* isolates poultry are still lacking in this region.

Therefore, the aim of this study was to investigate the prevalence of *mcr-1*-positive *E. coli* in diseased and healthy poultry across 11 cities in Jiangxi between 2020 and 2024. We characterized the molecular characteristics, AMR profiles, virulence gene repertoires, and pathogenic potential of these isolates. The findings of this study will provide baseline epidemiological data and a reference for the prevention and control of *mcr-1*-positive *E. coli* in local poultry, the rational use of antimicrobial agents, and the mitigation of public health risks.

## Materials and methods

### Strain sources

From 2020 to 2024, two groups of *E. coli* isolates were collected across 11 cities in Jiangxi. APEC (*n* = 291) were isolated from lesioned tissues (liver, heart, brain, etc.) of diseased poultry with typical colibacillosis signs (Tan et al., 2023; Tan M. F. et al., 2025). For the commensal fecal *E. coli* group, 350 fecal samples were collected from healthy poultry on 48 farms across the 11 cities. Fecal samples were directly streaked onto MacConkey agar plates (Hopebio, Qingdao, China) and incubated at 37 °C for 18 h. *E. coli*-like single colonies were confirmed following the protocols established in our previous research (Tan et al., 2023). Strains were cultured at 37 °C in lysogeny broth (LB) or on LB agar (Hopebio).

### Identification of *mcr-1*-positive strains

Colony PCR amplification was performed on avian pathogenic and avian fecal *E. coli* strains to detect the presence of the *mcr-1* gene. The sequences of the primer pair were as follows: AGTCCGTTTGTTCTTGTGGC (forward primer) and AGATCCTTGGTCTCGGCTTG (reverse primer). The PCR conditions were as follows: initial incubation at 96 °C for 8 min, followed by 30 cycles of denaturation at 95 °C for 30 s, annealing at 58 °C for 30 s, extension at 72 °C for 30 s, and a final extension at 72 °C for 5 min. The addition of ddH_2_O instead of bacterial DNA served as the negative control. Aliquots were analyzed by electrophoresis on a 0.8% (w/v) agarose gel. Samples showing a fragment size of 320 bp were determined as *mcr-1*-positive. The collection time, location, and other relevant details for each *mcr-1*-positive strain are listed in Supplementary Table S1.

### Minimum inhibitory concentration test

MIC tests were performed using commercial MIC test panels for enterobacteria (Meihua Medical Technology Co., Ltd., Zhuhai, China). The test panel contained the following 16 antimicrobial agents: Ampicillin (AMP), Amoxicillin/Clavulanic Acid (AMC), Ceftiofur (FUR), Ceftazidime (CAZ), Meropenem (MRP), Gentamicin (GM), Apramycin (AP), Spectinomycin (SPT), Tetracycline (TET), Florfenicol (FFC), Sulfisoxazole (SIZ), Sulfamethoxazole/Trimethoprim (SMZ-TMP), Enrofloxacin (ENR), Ofloxacin (OFX), Mequindox (MEQ), and Colistin (CST) (Supplementary Table S2). Experiments were performed following the manufacturer’s instructions. Briefly, a single colony of *E. coli* from a pure culture was inoculated into a vial of diluent. The bacterial suspension was adjusted to a 0.5 McFarland standard by comparison with a standard turbidity tube. Each well of the test panel was inoculated with 50 μL of bacterial suspension and 50 μL of cation-adjusted Mueller-Hinton broth (Hopebio). Negative control wells were filled with 100 μL of cation-adjusted Mueller-Hinton broth. After incubation at 37 °C for 18 h, the test panels were read using the MA120 Microbial Identification and Antimicrobial Susceptibility Testing System (Meihua). *E. coli* ATCC 25922 was used as the reference strain for quality control. The results were interpreted following the standardized protocol recommended by the Clinical and Laboratory Standards Institute (Supplementary Table S2) (James et al., 2023).

### Whole-genome sequencing and analysis

The *mcr-1*-positive *E. coli* strains were cultured to the mid-log phase (optical density at 600 nm ~ 0.8). Genomic DNA was extracted using the MiniBEST Bacteria Genomic DNA Extraction Kit (TaKaRa, Dalian, China) according to the manufacturer’s instructions. Whole-genome sequencing was performed on the Illumina MiSeq platform at Sangon Biotech (Shanghai) Co., Ltd., China. The sequencing reads were assembled, evaluated, and annotated as described previously (Tan J. et al., 2025). The complete genome sequences have been deposited in GenBank, and the accession numbers are listed in Table 1 and Supplementary Table S1. The genotype, including O serotype, H serotype, and multilocus sequence typing (MLST), was determined by uploading the whole-genome sequence to the Center for Genomic Epidemiology website^1^ and performing online analysis. MLST is based on the allelic profiles of seven conserved housekeeping genes: *adk*, *fumC*, *gyrB*, *icd*, *mdh*, *purA*, and *recA* (Song et al., 2025). The acquired antibiotic resistance genes and their genomic locations, including plasmid or chromosome with exact coordinates, were identified using ResFinder platform (version 4.4.3) and further verified via PlasmidFinder (version 2.0.1). Virulence genes were predicted using the VirulenceFinder platform (version 2.0.5). All parameters were set to default values.

**Table 1:** Characteristics of the *mcr-1*-positive *E. coli* strains in this study.

Strain	Host	Sample origin	Accession number	O serotype	Localization of *mcr-1*	*mcr-1* associated MGE
APEC-30	Duck	Tissue	SAMN41661682	O145	Plasmid IncX4	–
APEC-42	Duck	Tissue	SAMN41661694	O92	Plasmid IncI2	ISEc9
APEC-77	Duck	Tissue	SAMN41661729	O2/O50	Chromosome	ISApl1, cn_4749_ISApl1
APEC-78	Duck	Tissue	SAMN41661730	O23	Chromosome	–
APEC-79	Duck	Tissue	SAMN41661731	O2/O50	Chromosome	ISApl1, cn_4749_ISApl1
APEC-81	Duck	Tissue	SAMN41661733	O2/O50	Chromosome	ISApl1, cn_4749_ISApl1
APEC-82	Duck	Tissue	SAMN41661734	O103	Chromosome	IS682
APEC-91	Duck	Tissue	SAMN41661743	O17/O44/O77	Chromosome	–
APEC-180	Duck	Tissue	SAMN42691625	O101	Plasmid IncX4	–
APEC-181	Duck	Tissue	SAMN42691626	O17/O77	Plasmid p0111	–
APEC-182	Duck	Tissue	SAMN42691627	O101	Plasmid IncX4	–
APEC-183	Duck	Tissue	SAMN42691628	O101	Plasmid IncX4	–
APEC-184	Duck	Tissue	SAMN42691629	O23	Plasmid IncI2	ISSen6
APEC-185	Duck	Tissue	SAMN42691630	O9	Plasmid IncHI2	ISEc9, ISEc36
APEC-187	Duck	Tissue	SAMN42691632	O131	Plasmid IncI2	–
EAC-01	Duck	Feces	SAMN52752341	O174	Chromosome	ISApl1
EAC-02	Chicken	Feces	SAMN52752342	–	Plasmid IncI2	–
EAC-03	Chicken	Feces	SAMN52752343	O5	Plasmid IncI2	ISSen6
EAC-04	Chicken	Feces	SAMN52752344	–	Plasmid IncI2	–
EAC-05	Chicken	Feces	SAMN52752345	O131	Plasmid IncI2	–
EAC-06	Chicken	Feces	SAMN52752346	–	Plasmid IncI2	ISVsa5
EAC-07	Chicken	Feces	SAMN52752347	–	Plasmid IncI2	–
EAC-08	Chicken	Feces	SAMN52752348	O131	Plasmid IncI2	–
EAC-09	Duck	Feces	SAMN52752349	O9/O9a	Plasmid IncX4	–

### Construction of phylogenetic tree

Pangenome analysis was performed using Roary v3.13.0, which produced a concatenated alignment of 3,015 core genes. A maximum-likelihood phylogenetic tree was constructed from this alignment using RAxML v8.2.12 (Stamatakis, 2014) with the GTR + GAMMA substitution model and 1,000 bootstrap replicates. The tree was visualized using iTOL v5. All parameters were set to default values.

### Pathogenicity assays

*Galleria mellonella* was used as the animal model to evaluate the virulence of *mcr-1*-positive *E. coli* (Cutuli et al., 2019). Worms weighed between 0.55 and 0.65 g at the time of inoculation. Strains at the mid-log phase were collected, washed, and adjusted with normal saline to an optical density at 600 nm of ~0.19 (high dose) or ~0.09 (low dose). LB agar plate counting confirmed that the two bacterial suspensions contained approximately 2 × 10^8^ CFU/mL and 1 × 10^8^ CFU/mL, respectively. Eight worms per group were each injected with 25 μL of inoculum into the lower left proleg using a BD insulin syringe (New Jersey, United States). Worms were incubated at 37 °C in sterile plastic culture plates (9 cm) without food for up to 3 days. The same volume of the previously studied O145 virulent strain NC22 served as the positive control (Tan et al., 2024), while an equivalent amount of sterile saline served as the negative control. Survival rates were recorded at 1-day intervals.

## Results

### Prevalence of *mcr-1*-positive strains

In this study, a total of 591 *E. coli* strains were used for the identification of *mcr-1.* Among these, 291 strains were isolated from diseased poultry tissues during our previous epidemiological investigations (Tan et al., 2023; Tan M. F. et al., 2025). The remaining 300 strains were obtained from the feces of healthy poultry. PCR analysis revealed that the *mcr-1* gene was detected in 24 strains, corresponding to an overall detection rate of 4.06% (24/591). Among the positive strains, 15 were derived from the diseased poultry group (5.15%, 15/291) and were identified as APEC, whereas the remaining 9 positive strains were isolated from healthy poultry feces (3.00%, 9/300) (Table 1). Moreover, the APEC strains carrying *mcr-1* were predominantly isolated from the livers of diseased ducks, while the *mcr-1*-positive intestinal *E. coli* strains were mainly recovered from the intestines of healthy chickens (Supplementary Table S1).

### Antimicrobial susceptibility profiles

**Figure 1:**
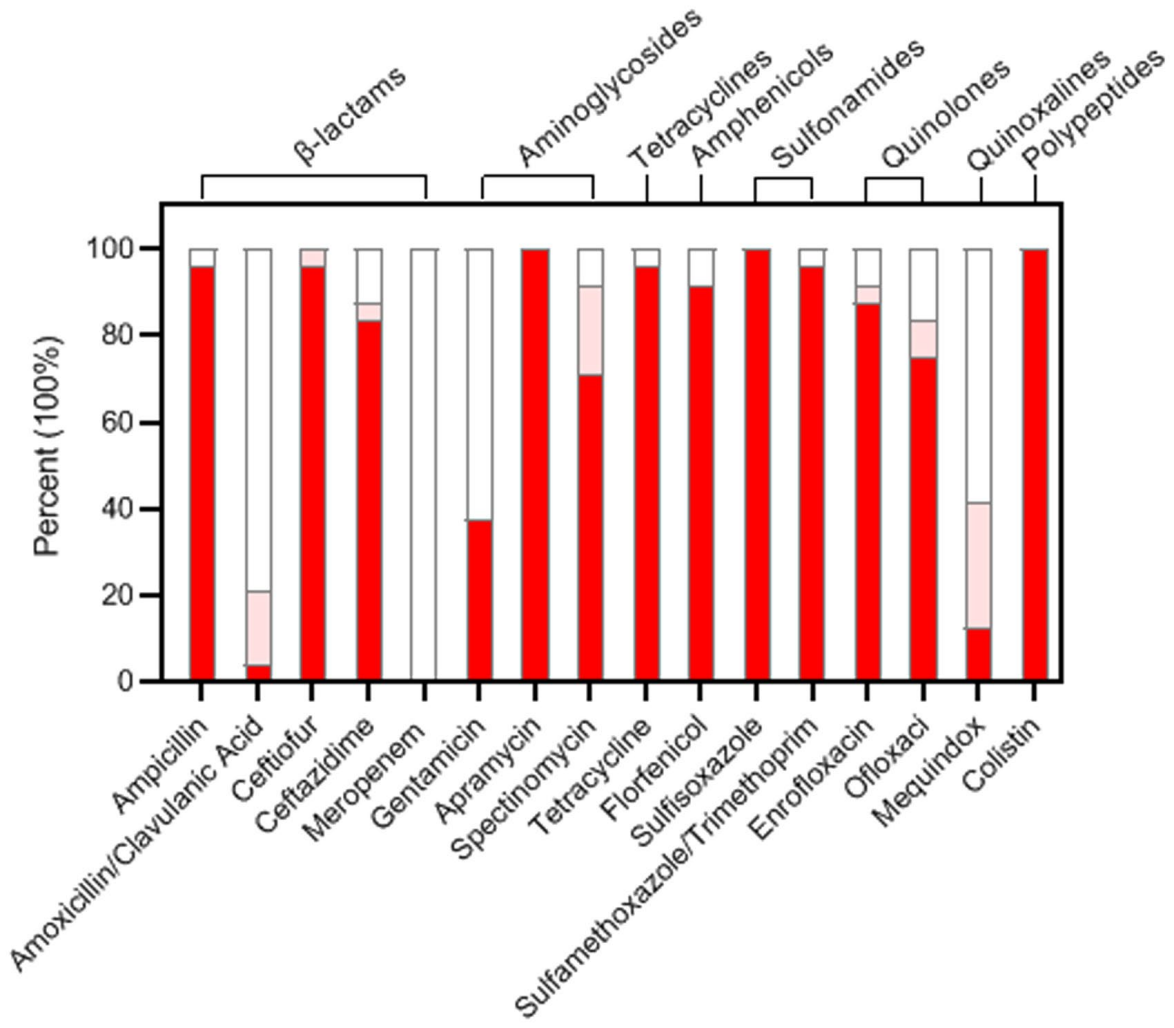
Antimicrobial resistance profiles of the *mcr-1*-positive *E. coli* isolates.

The MIC results for the 24 *mcr-1*-positive strains are presented in Supplementary Table S3 and Figure 1. All 24 strains exhibited resistance to CST, with MIC values ranging from 4 to 8 μg/mL. High resistance rates were observed for multiple antimicrobial agents: AP and SIZ both showed 100.00% resistance, followed by AMP, FUR, TET, and SMZ-TMP (95.83% each). ENR and CAZ also demonstrated notably high resistance rates of 87.50 and 83.33%, respectively. In contrast, all strains remained susceptible to MRP. Other agents with relatively higher susceptibility rates included AMC at 79.17% and GM at 62.50%. Notably, MDR, defined as resistance to at least three classes of antimicrobial agents, was observed in all 24 strains (100.00%).

### Phylogenetic analysis

**Figure 2:**
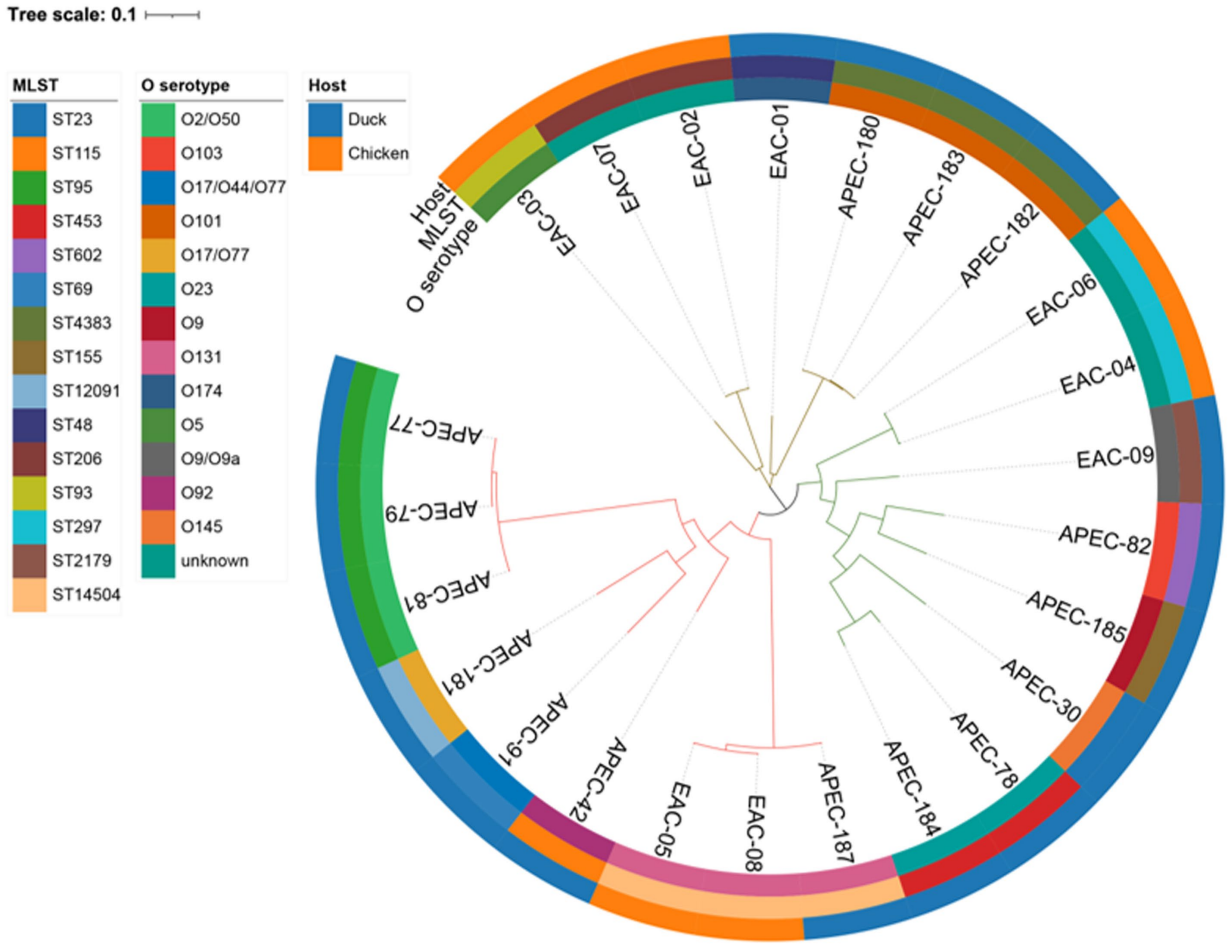
Phylogenetic tree based on core genes of the *E. coli* strains.

Phylogenetic analysis was performed to assess the genetic relatedness of the *E. coli* isolates, and the resulting phylogenetic tree is presented in Figure 2. The tree revealed a clear genetic segregation pattern, with three distinct major clades observed.

The first clade (highlighted in red) consisted exclusively of intestinal *E. coli* isolates (EAC-02, EAC-03, EAC-07), indicating a close genetic relationship among these strains. The second clade (highlighted in green) comprised one intestinal *E. coli* isolate (EAC-01) and three APEC isolates (APEC-180, APEC-182, APEC-183). The third and largest clade (highlighted in purple) contained the majority of isolates, including both intestinal *E. coli* (EAC-04, EAC-05, EAC-06, EAC-08, EAC-09) and APEC strains, demonstrating a high degree of genetic diversity within this group. Notably, no strict source-specific clustering was observed in the large purple clade, as intestinal *E. coli* and APEC isolates were interspersed throughout multiple subclades. This finding indicates that while some isolates exhibit source-associated genetic relatedness, others share common genetic lineages regardless of their origin.

### Molecular characteristics of *mcr-1*-positive *Escherichia coli* strains

Whole-genome sequencing and bioinformatics analysis were performed on the *mcr-1*-positive isolates to determine their genotypic profiles. MLST identified 15 distinct sequence types (STs), among which ST95, ST4383, and ST14504 were the most prevalent, each accounting for 3 out of 24 isolates (12.50%) (Supplementary Table S1). O serotyping revealed 13 different O antigens, with O101, O131, and O2/O50 being the most common (each 3/24, 12.50%), while four strains were non-typeable (Table 1). The remaining STs and O serotypes were detected in only one or two isolates each. Both typing methods indicated a dispersed distribution, with no single dominant ST or O serotype, suggesting that the dissemination of *mcr-1* in this region occurred across diverse genetic backgrounds.

### Genotypic antimicrobial resistance profiling

All isolates harbored the *mcr-1* gene, consistent with the colistin-resistant phenotype. Genomic analysis revealed diverse genetic contexts underlying *mcr-1* localization and associated mobile genetic elements (MGEs; Table 1). Among the 24 isolates characterized, *mcr-1* was predominantly located on plasmids, with IncI2 as the most frequent replicon type, followed by IncX4, IncHI2, and p0111. Chromosomal integration of *mcr-1* was detected in eight isolates, most of which belonged to the APEC group. A range of insertion sequences were found in association with *mcr-1*, including ISApl1, ISEc9, ISSen6, IS682, ISEc36, and ISVsa5. Several isolates carried composite transposon-like structures such as cn_4749_ISApl1, whereas some strains carried *mcr-1* on either plasmids or the chromosome without recognizable flanking MGEs.

Sequence alignment analysis was performed on the 24 *mcr-1* genes identified in this study, using the *mcr-1* sequence from strain KP347127 as a reference. The results showed that 21 isolates shared 100% sequence identity with the reference *mcr-1*, while the remaining three isolates (APEC-180, APEC-182, and APEC-183) exhibited 99.94% sequence similarity to the reference (Supplementary Table S1). All core *α*-helices (α1–α17), *β*-sheets (*β*1–*β*11), and *η*-helices (η1–η7) were completely conserved, and single amino acid polymorphisms were located in the flexible loop regions of non-core functional domains, indicating that the catalytic activity and membrane localization of the protein remain unaffected (Supplementary Figure S1).

**Figure 3:**
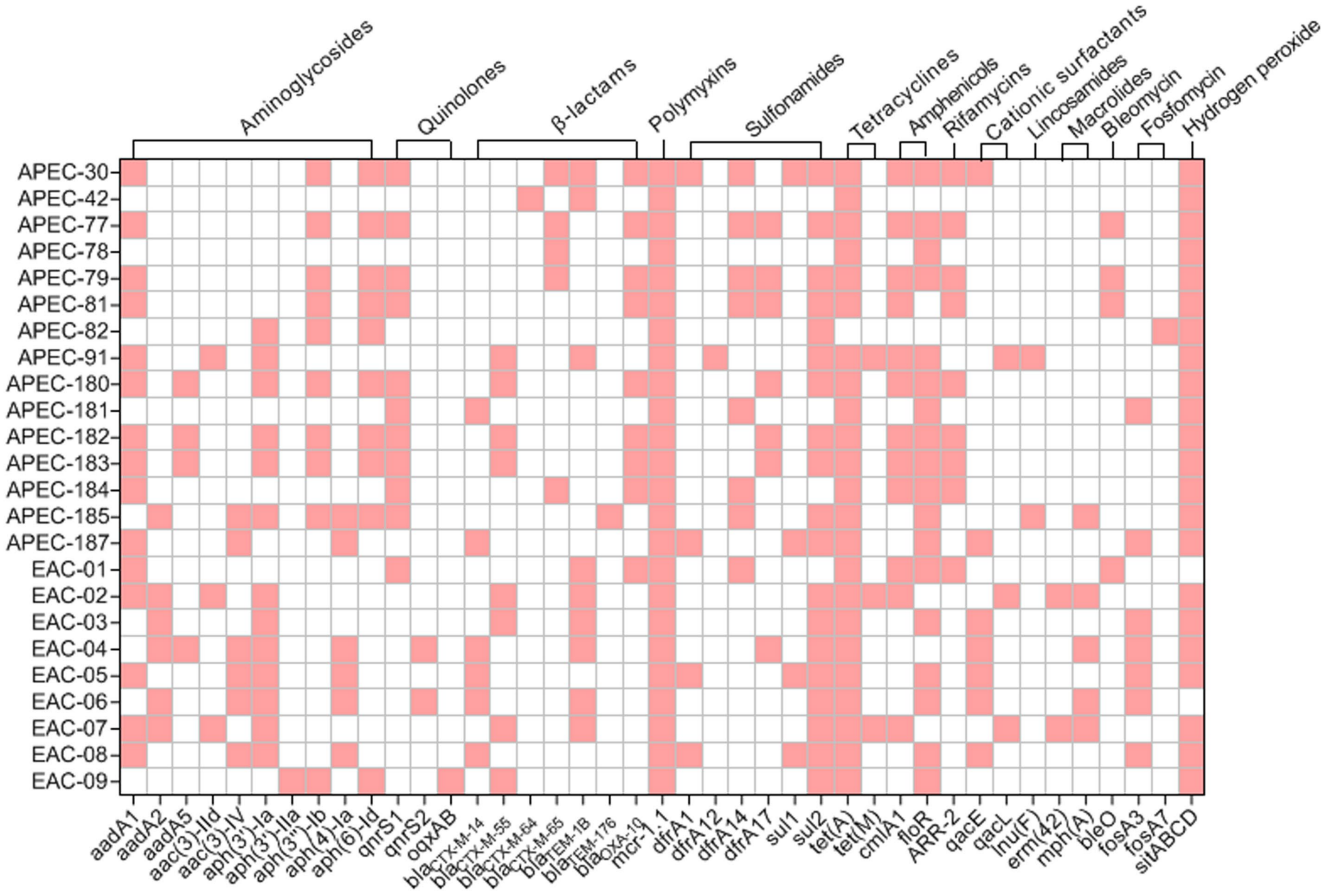
Distribution matrix of acquired antibiotic resistance genes in the *E. coli* strains. White indicates absence, and pink indicates presence of the corresponding gene.

The number of acquired resistance genes per isolate ranging from 5 to 18 (mean = 13.4) (Supplementary Table S1; Figure 3). Among these, 15 APEC isolates carried 5–18 resistance genes (mean = 12.9), while 9 intestinal *E. coli* isolates carried 10–17 resistance genes (mean = 14.2), indicating a higher resistance gene burden in intestinal *E. coli* strains. The highest number of resistance genes was detected in APEC-30 (18 genes), whereas APEC-42 and APEC-78 carried the fewest (5 genes each). A variety of resistance determinants mediating non-susceptibility to tested antimicrobials were detected, including *bla*_TEM_ (*β*-lactams), *aph*(3′)-IIa (aminoglycosides), *tetA/tetB* (tetracyclines), *floR* (phenicols), *sul1*/*sul2*/*sul3* (sulfonamides), and *qnrS*/*oqxA* (fluoroquinolones). The most prevalent resistance genes were *tet(A)* (95.3%, 23/24), *bla*_CTX-M_ (83.33%, 20/24), *sul2* (79.16%, 19/24), and *floR* (75.00%, 18/24). The *bla*_CTX-M_ genes consisted of four variants: *bla*_CTX-M-14_, *bl*_*a*CTX-M-55_, *bla*_CTX-M-64_, and *bla*_CTX-M-65_. Overall, the extensive carriage and wide distribution of resistance determinants revealed a notable multidrug-resistance phenotype among these *mcr-1*-positive poultry-derived *E. coli* isolates, highlighting a potential risk of transmission and the challenges for clinical treatment.

### Virulence genotypic characteristics

**Figure 4:**
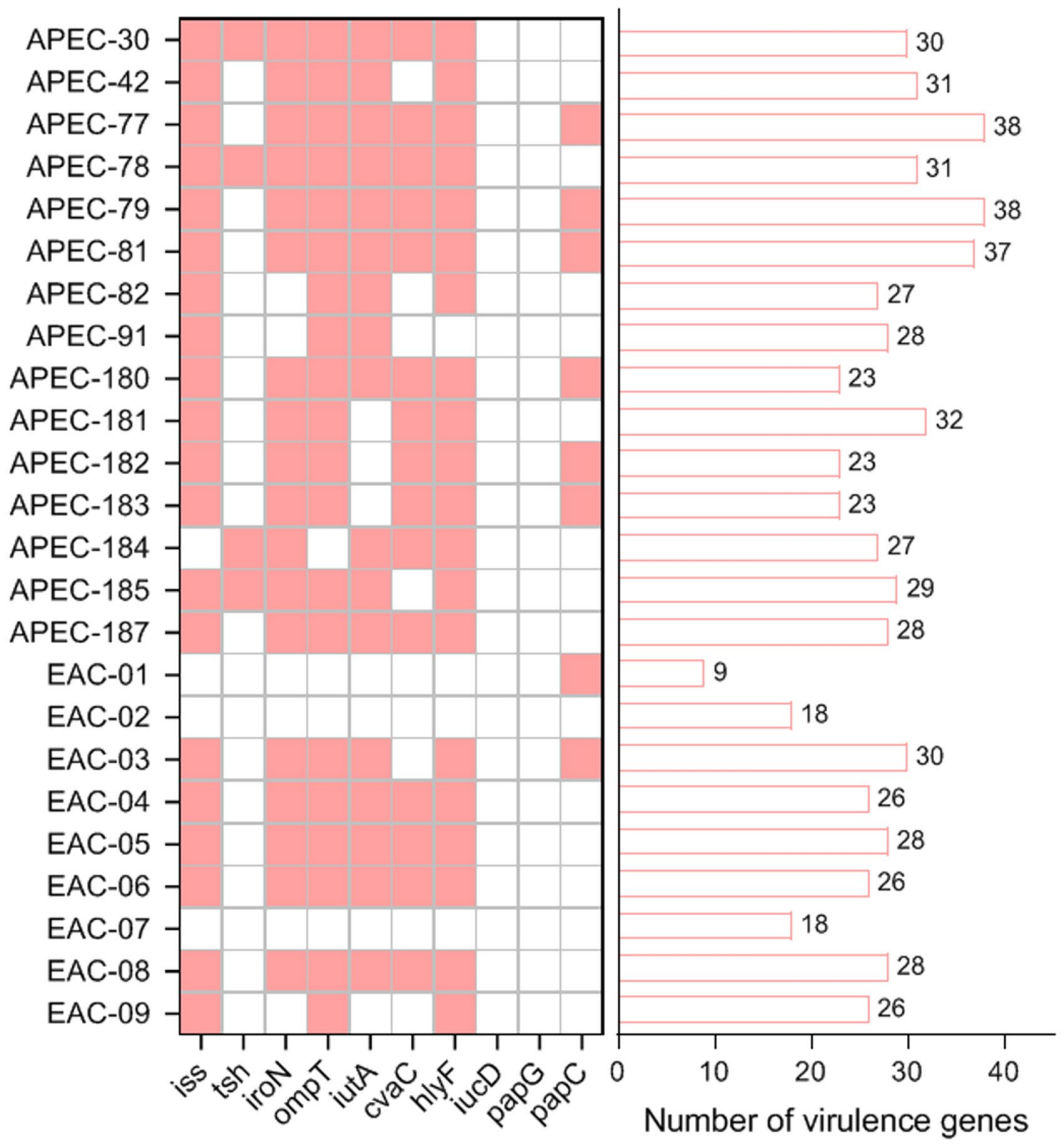
Distribution matrix of virulence-associated genes in the *E. coli* strains. Left panel: distribution of 10 key virulence-associated genes. Right panel: number of virulence factors carried by each strain.

Previous studies have recognized ten key virulence-associated genes, including *iss*, *tsh*, *iroN*, *ompT*, *iutA*, *cvaC*, *hlyF, iucD*, *papG* allele (II/III), and *papC*, which are more frequently detected in APEC isolates than in nonpathogenic *E. coli* (Ovi et al., 2023). We summarized the distribution of these ten virulence genes among the 24 bacterial isolates (Figure 4). The virulence factors *iss* (87.50%, 21/24), *ompT* (87.50%, 21/24), *hlyF* (83.33%, 20/24), *iroN* (75.00%, 18/24), and *iutA* (70.83%, 17/24) were detected at high prevalence. In contrast, *iucD* and *papG* were not detected in any of the isolates. In addition, the *sitABCD* operon, which mediates hydrogen peroxide tolerance and promotes host immune evasion, was present in the majority of isolates (91.67%, 22/24; Figure 3), representing a conserved adaptive trait in this *mcr-1*-positive *E. coli* population.

Quantitative analysis of virulence factor carriage revealed a wide range in the number of virulence genes detected per isolate (Figure 4), spanning from 23 to 38 among APEC strains (mean = 29.7). APEC-77 and APEC-79 harbored the most extensive virulomes, with 38 virulence genes each. In contrast, APEC-180, APEC-182, and APEC-183 exhibited the most restricted virulence gene repertoires, with only 23 genes each, suggesting reduced virulence capacity. Compared with APEC isolates, intestinal *E. coli* strains generally carried fewer virulence determinants, with counts ranging from 9 to 30 (mean = 23.2). Among these isolates, EAC-01 possessed only 9 virulence genes.

### Pathogenicity assessment

**Figure 5:**
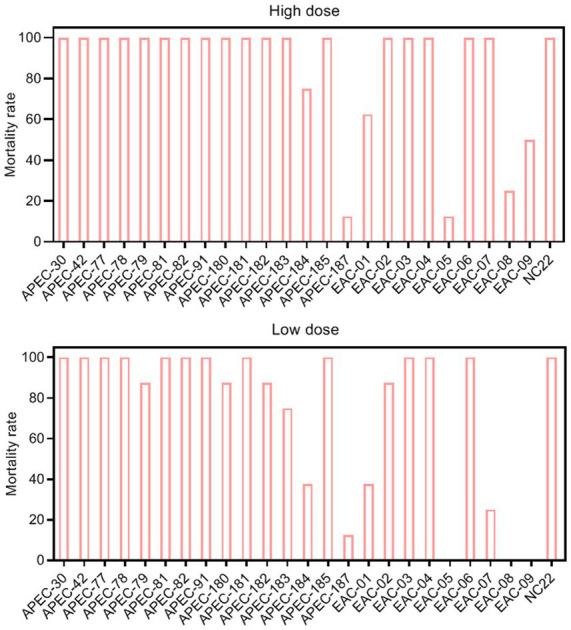
Results of the challenge test. Top panel: mortality of *Galleria mellonella* challenged with the high dose. Bottom panel: mortality of *G. mellonella* challenged with the low dose.

The pathogenic potential of the isolates was evaluated using *G. mellonella* challenge assays at high and low inoculum doses, with the resulting mortality rates presented in Supplementary Table S4 and Figure 5. At the high dose, 18 isolates caused 100% mortality in the worms, including 13 APEC strains and five intestinal *E. coli* strains. Among these, 12 isolates were capable of inducing 100% mortality even at the low dose, comprising eight APEC strains and four intestinal *E. coli* strains. Interestingly, APEC-187, EAC-05, EAC-08, and EAC-09 carried 28, 28, 28, and 26 virulence factors, respectively, but did not exhibit high virulence. IIn contrast, EAC-02 and EAC-07, which carried only 18 virulence factors, caused 100% mortality in the worms at the high dose. These results suggest that the number of virulence genes alone cannot serve as the sole indicator of pathogenicity in this infection model.

## Discussion

In this study, we systematically characterized the genomic and phenotypic features of 24 *mcr-1*-positive *E. coli* isolates collected from diseased and healthy poultry across Jiangxi between 2020 and 2024. The overall prevalence of *mcr-1* was 4.06%, with a higher detection rate in diseased poultry (5.15%) than in healthy ones (3.00%). This is consistent with the notion that clinical infection pressure from host immune responses and antibiotic treatment drives the selection and enrichment of resistant strains within infected hosts (Kent et al., 2025; Tyne et al., 2019). Geographically, the prevalence in Jiangxi was higher than that reported in Hebei (2.02%), Uganda (1.2%) (Wainaina et al., 2025) and Algeria (3.05%), but lower than in Shandong (15.83%) (Li et al., 2025) and Pakistan (14.00%) (Umair et al., 2023), highlighting the heterogeneous distribution of *mcr-1*-positive *E. coli* across regions.

All 24 isolates exhibited colistin resistance and 100% MDR, with high resistance rates to AP, SIZ, AMP, FUR, TET, and SMZ-TMP (all >95%), while remaining fully susceptible to MRP. The resistance profile was consistent with the presence of acquired resistance genes, including *tet(A)*, *bla*_CTX-M_, *sul2*, and *floR*. The high prevalence of resistance to aminoglycosides, sulfonamides, and colistin is particularly concerning, as these agents are commonly used in veterinary medicine (Roth et al., 2019). The extensive carriage of resistance genes in intestinal isolates underscores the hidden threat posed by apparently healthy poultry as reservoirs of MDR bacteria, which may enter the food chain and contribute to the spread of AMR to humans (Woyda et al., 2023). Therefore, integrated strategies, including routine resistance surveillance, prudent antimicrobial use, and alternative therapeutic approaches, are urgently needed to mitigate these risks.

Molecular localization analysis revealed that *mcr-1* was predominantly located on plasmids, with IncI2 being the most common replicon type, followed by IncX4, IncHI2, and p0111. This predominance of IncI2 is consistent with previous studies identifying it as a primary conjugative vector driving *mcr-1* dissemination in food-producing animals in China (Yin et al., 2021; Wang et al., 2020). Chromosomal integration of *mcr-1* was detected in eight isolates, mainly APEC strains. Chromosomally encoded *mcr-1* is generally more stable and less likely to be lost than plasmid-borne copies, which may promote the persistent maintenance of colistin resistance even in the absence of direct selection pressure (Shen et al., 2020). This combination of plasmid-mediated mobility and chromosomal stability may enhance the long-term survival of *mcr-1* in poultry-associated *E. coli* populations.

Virulence genotyping revealed a high prevalence of APEC-associated determinants involved in iron acquisition (*iroN*, *iutA*), serum resistance (*iss*), and cytotoxicity (*ompT*, *hlyF*), suggesting that these strains possess the genetic capacity to cause infections in poultry. However, classical virulence genes such as the fimbrial adhesin-encoding gene *papG* and the siderophore receptor gene *iucD* (Ovi et al., 2023) were absent in all isolates. This finding highlights the difficulty of defining a core set of APEC virulence markers, and supports the possibility that alternative factors may compensate for the absence of these commonly recognized genes.

In the *G. mellonella* infection assay, most isolates exhibited strong lethality, with some intestinal commensal strains also causing high mortality, indicating that these isolates may pose a zoonotic risk. Notably, virulence gene count did not strictly correlate with lethal ability. For example, strains with fewer virulence determinants sometimes caused complete mortality, while others with more genes showed only moderate virulence. This discrepancy reflects both model limitations and the complexity of virulence determination. The *G. mellonella* model relies on innate immunity, and its hemocoel injection route avoids the natural mucosal barriers. In virulence determination, pathogenicity depends on gene expression levels, functional integrity of virulence factors, synergistic interactions, and host environmental conditions (Jackson et al., 2024). These findings suggest that virulence gene profiling alone is insufficient for predicting pathogenic outcomes, and functional assays are essential for a comprehensive virulence assessment.

Phylogenetic analysis revealed no strict source-specific clustering between APEC and commensal isolates, with strains of different origins sharing close genetic relatedness across multiple subclades. This supports the hypothesis that intestinal commensal *E. coli* serve as a virulence gene reservoir and evolutionary precursor of APEC. Commensal strains may acquire specific virulence determinants via horizontal gene transfer and evolve into pathogenic lineages capable of causing systemic infections (Song et al., 2025). The concurrent carriage of AMR genes in these commensal isolates further highlights the risk of commensal-to-pathogen transition, posing a combined threat to poultry production and public health.

Several limitations of the present study should be acknowledged. First, the small number of *mcr-1*-positive isolates (*n* = 24) limits the strength of regional epidemiological inferences. Larger, multi-center longitudinal studies are needed to confirm our findings. Second, although the *G. mellonella* model is a well-established surrogate for evaluating bacterial virulence, it cannot fully replicate the complex host–pathogen interactions that occur during natural infections in avian or mammalian hosts (Tsai et al., 2016). Third, our genotypic characterization lacked experimental validation (e.g., gene expression, phenotypic assays). Fourth, no conjugation or transformation experiments were performed to validate the horizontal transfer of *mcr-1*-carrying elements. Genomic predictions require functional confirmation. Accordingly, future studies with expanded sample sizes, functional assays, and improved animal models are warranted to confirm our findings and elucidate the molecular basis of the observed genotype–phenotype discrepancies.

## Conclusion

This study represents a systematic investigation of *mcr-1*-positive *E. coli* across 11 cities in Jiangxi. It provides preliminary epidemiological baseline data on 24 positive isolates from poultry, revealing notable prevalence, extensive MDR, genetic diversity, and pathogenic potential among the tested strains. Both diseased and healthy poultry serve as important reservoirs of *mcr-1*, with intestinal *E. coli* playing an important role in persistence of resistance genes. The coexistence of plasmid-borne and chromosome-integrated *mcr-1* genomically suggests a dual maintenance mechanism with putative transmissibility that may sustain colistin resistance despite the national ban, which requires further functional validation. Notably, highly pathogenic APEC strains carried a high burden of MDR genes, while some intestinal commensal *E. coli* from healthy poultry also exhibited strong lethality in the *G. mellonella* model, indicating a potential dual threat to animal and public health that warrants further large-scale investigation. These findings provide preliminary molecular epidemiological data and a theoretical basis for controlling colistin-resistant bacteria in local poultry production, promoting rational antimicrobial use, and reducing public health risks. Expanded surveillance, standardized APEC control, and restricted spread of resistant bacteria along the food chain are recommended to safeguard animal and public health.

## Data Availability

The datasets presented in this study can be found in online repositories. The names of the repository/repositories and accession number(s) can be found in the article/Supplementary material.

## Footnotes

1
http://www.genomicepidemiology.org/services/

## Supplementary material

SUPPLEMENTARY FIGURE S1 Multiple sequence alignment of the *mcr-1* genes. The sequences used here were collected from plasmid pHNSHP45 (GenBank accession no. KP347127), APEC030 (accession no. SAMN41661682), and APEC180 (accession no. SAMN42691625).
