# Supplementary figures and images for: Molecular epidemiology and pathogenic potential of mcr-1-positive Escherichia coli isolated from healthy and diseased poultry in Jiangxi, China
Source: Front Microbiol. 2026 Jul 7;17:1855169. doi: 10.3389/fmicb.2026.1855169 (PMC13385662; doi:10.3389/fmicb.2026.1855169)

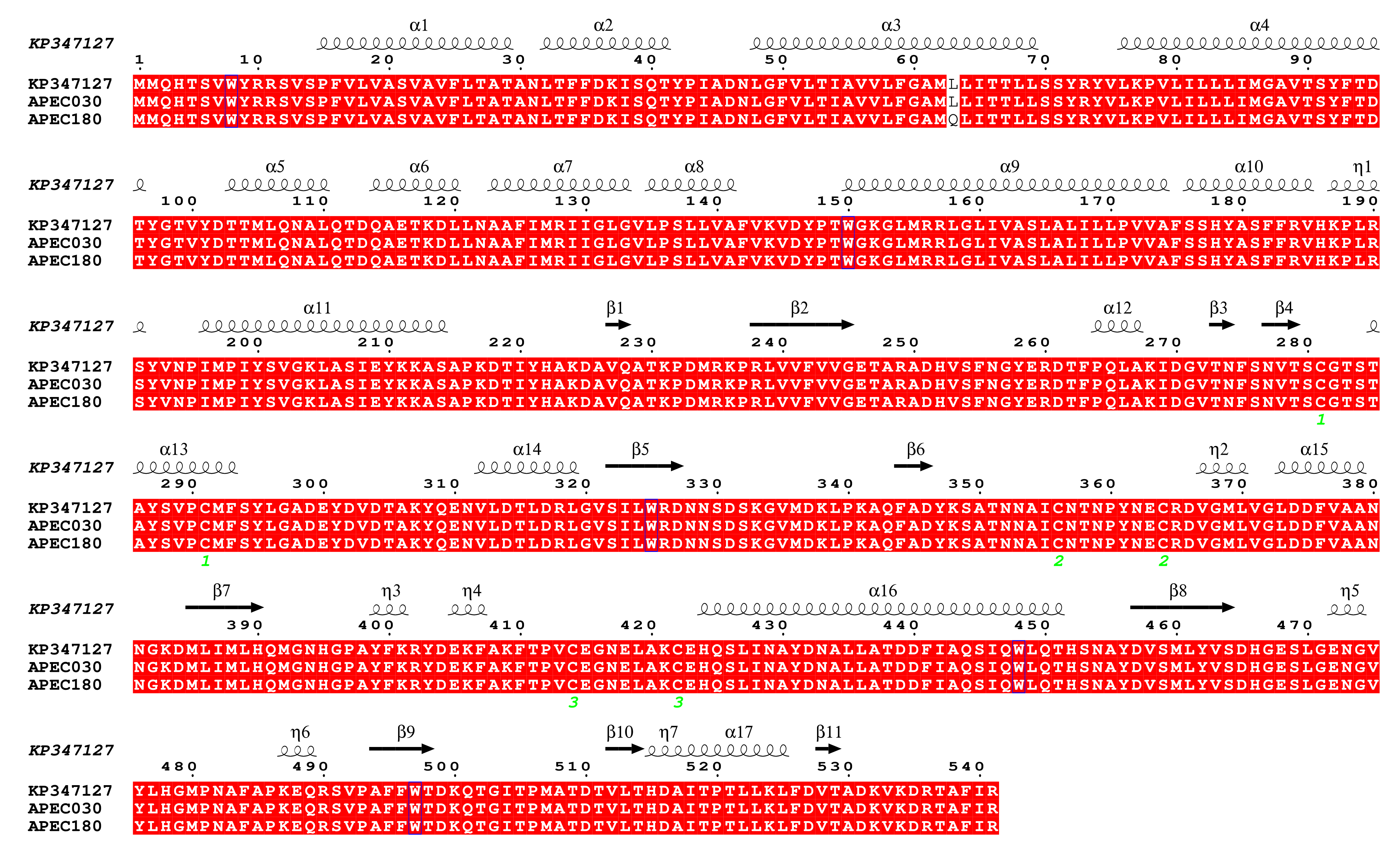

Supplement: SUPPLEMENTARY FIGURE S1 — Multiple sequence alignment of the mcr-1 genes. The sequences used here were collected from plasmid pHNSHP45 (GenBank accession no. KP347127), APEC030 (accession no. SAMN41661682), and APEC180 (accession no. SAMN42691625). [file Image_1.TIF]
