# Supplementary material for: Molecular epidemiology and pathogenic potential of mcr-1-positive Escherichia coli isolated from healthy and diseased poultry in Jiangxi, China
Source: Front Microbiol. 2026 Jul 7;17:1855169. doi: 10.3389/fmicb.2026.1855169 (PMC13385662; doi:10.3389/fmicb.2026.1855169)
Supplement: Supplementary file 3 [file Table_2.DOCX]

Table S2**.** Specific characteristics of each antimicrobial used in this study.

| Category | Antimicrobial | **Concentration range** (μg/mL) | **MIC breakpoints (μg/mL)** | | |
| --- | --- | --- | --- | --- | --- |
|  |  |  | Resistant | Intermediate | Susceptible |
| β-lactams | Ampicillin | 0.25–512 | ≤ 8 | – | ≥ 32 |
|  | Amoxicillin/Clavulanic Acid | 0.25/0.12–512/256 | ≤ 8/4 | 16/8 | ≥ 32/16 |
|  | Ceftiofur | 0.12**–**256 | ≤ 2 | 4 | ≥ 8 |
|  | Ceftazidime | 0.12**–**256 | ≤ 4 | 8 | ≥ 16 |
|  | Meropenem‌ | 0.0075–16 | ≤ 1 | 2 | ≥ 4 |
| Aminoglycosides | Gentamicin | 0.25**–**512 | ≤ 4 | 8 | ≥ 16 |
|  | Apramycin | 0.06–128 | ≤ 4 | – | ≥ 8 |
|  | Spectinomycin | 0.25**–**512 | ≤ 16 | 32 | ≥ 64 |
| Tetracyclines | Tetracycline | 0.25**–**512 | ≤ 4 | 8 | ≥ 16 |
| Amphenicol | Florfenicol | 0.25**–**512 | ≤ 8 | 16 | ≥ 32 |
| Sulfonamides | Sulfisoxazole | 0.25**–**512 | ≤ 256 | – | ≥ 512 |
|  | Sulfamethoxazole/Trimethoprim | 0.06/0.2–32/608 | ≤ 2/38 | – | ≥ 4/76 |
| Quinolones | Enrofloxacin | 0.015–32 | ≤ 0.25 | 0.5 | ≥ 1 |
|  | Ofloxacin | 0.03–64 | ≤ 1 | 2 | ≥ 4 |
| Quinoxalines | Mequindox | 1–512 | ≤ 8 | 16 | ≥ 32 |
| Polypeptides | Colistin | 0.12–256 | ≤ 2 | – | ≥ 4 |
